# Stakeholders’ perspectives on the post-mortem use of genetic and health-related data for research: a systematic review

**DOI:** 10.1038/s41431-019-0503-5

**Published:** 2019-09-16

**Authors:** Marieke A. R. Bak, M. Corrette Ploem, Hakan Ateşyürek, Marieke T. Blom, Hanno L. Tan, Dick L. Willems

**Affiliations:** 1 Section of Medical Ethics, Department of General Practice, Amsterdam UMC, University of Amsterdam, Amsterdam, The Netherlands; 2 Section of Health Law, Department of Social Medicine, Amsterdam UMC, University of Amsterdam, Amsterdam, The Netherlands; 3 Faculty of Medicine, Amsterdam UMC, University of Amsterdam, Amsterdam, The Netherlands; 4 Department of Cardiology, Heart Center, Amsterdam UMC, University of Amsterdam, Amsterdam, The Netherlands

**Keywords:** Medical ethics, Ethics, Genetic testing, Genetics research, Health policy

## Abstract

The majority of biobank policies and consent forms do not address post-mortem use of data for medical research, thus causing uncertainty after research participants’ death. This systematic review identifies studies examining stakeholders’ perspectives on this issue. We conducted a search in MEDLINE, CINAHL, EMBASE and Web of Science. Findings were categorised in two themes: (1) views on the use of data for medical research after participants’ death, and (2) perspectives regarding the post-mortem return of individual genetic research results. An important subtheme was the appropriate authority and degree of control over posthumous use of data. The sixteen included studies all focused on genetic data and used quantitative and qualitative methods to survey perspectives of research participants, family members, researchers and Institutional Review Board members. Acceptability of post-mortem use of data for medical research was high among research participants and their relatives. Most stakeholders thought participants should be informed about post-mortem research uses during initial consent. Between lay persons and professionals, disagreement exists about whether relatives should receive actionable genetic findings, and whether the deceased’s previous preferences can be overridden. We conclude that regulations and ethical guidance should leave room for post-mortem use of personal data for research, provided that informed consent procedures are transparent on this issue, including the return of individual research findings to relatives. Future research is needed to explore underlying causes for differences in views, as well as ethical and legal issues on the appropriate level of control by deceased research participants (while alive) and their relatives.

## Introduction

Progress in medical science is promoted by the availability, sharing and use of vast amounts of personal (i.e., identifiable) data from patients and healthy volunteers [1–3]. In recent decades there has been a rise in the number of longitudinal studies and long-term biobanking projects. These extended timeframes increase the likelihood of a research participant passing away, which is especially true for studies with high mortality risks such as certain types of cancer or cardiac disease. However, it seems that the majority of study policies and consent forms do not address the use of data after death.

An international review of 54 biobank consent forms and information documents found that only two of these (one Canadian and one British biobank) discussed the fate of data after death [4]. Similarly, an analysis of 14 biobanks from different European countries found that only three (from Portugal, Luxembourg and the United Kingdom) mentioned this aspect in their consent forms [5], and our own exploration of ten European cardiac arrest registries and biobanks revealed that none of their consent forms specifically discussed post-mortem use of data [6]. Likewise, a German study analysed 30 biobank consent documents of which none made reference to procedures for dealing with data after participants’ death [7].

This lack of information may lead to incorrect assumptions among biobank participants about what happens to their data when they pass away. Moreover, in certain settings (e.g., medical emergencies) it is impossible to obtain prospective consent for the use of data for research, which causes uncertainty among researchers regarding the use of data when people die before consent could be obtained [8]. Furthermore, even if consent has been asked during the participant’s lifetime, this consent cannot be renewed for secondary uses of data, i.e., when substantial changes to the research project and its aims are made after the participant’s death.

In light of these issues, two main ethical questions arise. Firstly, it can be questioned whether post-mortem use and sharing of identifiable research data is ethically permissible at all. When research participants have not been asked to consent to post-mortem use of their data, does this mean that researchers should not be allowed to use the data, or, at the other end of the spectrum, that no restrictions are needed given the apparent impossibility of harm to the dead participant? This question is of particular significance in genetic research, because through its sensitive nature and unique identifiability, genetic research data may give rise to privacy and discrimination concerns among living relatives even after the initial data subject has passed away [9, 10]. However, the expanding availability of genome and exome sequencing analyses also generate a growing number of potentially clinically actionable findings that could be beneficial for blood relatives to know. Should these individual research findings be communicated to relatives of the deceased? A related question is whether it is desirable, and to what extent, that, through informed consent, research participants or their relatives are granted control over the post-mortem uses and sharing of data, including individual research results.

### Legal and ethical frameworks

Do international guidelines and regulations provide answers to the aforementioned ethical issues? A review of international legal and ethical guidance governing biobanking and genetic research showed that few guidelines directly address the situation where a research participant has died, and none provide specific recommendations about the return of individual genetic research results to deceased participants’ relatives [4]. The effect of death on participants’ initial consent is specifically mentioned only in a World Health Organization report, which recognises that deceased participants still have interests, by stating that “death of a sample source only affects the primacy of his/her interests, it does not extinguish them” and that ethics approval is needed to rebalance these interests after death (Recommendation No. 13) [11]. Yet, the focus of this recommendation is on samples and not data as such, and moreover it remains unclear how and by whom this balancing should be done.

Binding legislation on post-mortem use of research data is limited in detail [4]. In the United States, for example, the research use of deceased persons’ data is not regarded as *human subject* research, and data may be used post-mortem for research even if obtained outside of a consented research project [12]. Similarly, regarding the European perspective, the General Data Protection Regulation 2016/679 (GDPR) of the European Union (EU), that was implemented in May 2018, does not apply to deceased persons (see Recital 27) [13].

Moreover, legislation varies between nations. Some EU member states have independently enacted post-mortem data protection legislation, such as Estonia where under the former Personal Data Protection Act, consent from next of kin used to be required for the use of deceased participants’ data. This is changed, however, in a new version of the law: now, the prospective consent of the data subject suffices for the first 10 years after death [14], similar to the situation in Denmark [15]. EU countries that do still allow post-mortem control by relatives (e.g., in terms of access, rectification or deletion), unless expressly prohibited by the data subject while alive, include Italy [16], Spain [17], Slovakia [18] and Hungary [19]. This is in contrast with countries like the Netherlands [20], Belgium [21], Sweden [22] or Finland [23], where data protection regulation contains no special provisions regarding personal data of deceased persons.

Nevertheless, there seems to be agreement at least in Europe that data of deceased patients are still protected—that is, in principle, with deviations possible for research purposes—under doctors’ duty to medical confidentiality which continues to apply after death [24], and countries may have specific legislation regarding medical research that applies alongside data protection regulation. In the Netherlands, for example, the Data Protection Act [20] does not include provisions on post-mortem data, but the National Medical Treatment Act [25] allows that data be used for research when the person has given consent during his or her lifetime or when asking consent is no longer possible (after death). Thus, in the Netherlands relatives have no legal authority to decide on post-mortem use of already collected data from their deceased relative for research [26]. Data protection laws may also be supplemented by specific national legislation on the (post-mortem) return of individual findings. However, an international review suggests that binding legislation and case law may insufficiently address the issue of disclosing a deceased participant’s genetic findings to relatives [27].

### Aims and scope

As a result of the lack of clear international guidance, researchers, particularly those active in the field of genetics, struggle with how to handle deceased subjects’ data, especially when the decedent’s wishes are unknown [28, 29]. Our aim in this article is to provide an overview of the literature on participants’ and other stakeholders’ perspectives regarding post-mortem use of previously collected research data, in order to provide input (rather than draw normative conclusions) for study policies and legal or ethical guidelines. Therefore, we conducted a systematic search and synthesis of the empirical literature on this topic. In this paper, we discuss two themes that arose from reviewed studies: views and experiences regarding (a) the post-mortem use of genetic and health-related data for research and (b) the return of individual genetic research findings to relatives after the research participant’s death. An important subtheme for both questions was related to views regarding the appropriate authority and extent of control over these research data after death.

The scope of this review is limited to the post-mortem research with genetic (or broader: genomic) and other health-related data (contained in databases or biobanks) obtained *during the lifetime* of a research participant. We chose this focus because procurement of samples *after death* raises a qualitatively different set of ethical questions (e.g., concerns about body and burial), and because of the lack of guidance compared with posthumous collection [4, 30].

## Materials and methods

### Search strategy

We systematically searched for studies eligible for inclusion in this review using the following electronic databases: PubMed (including MEDLINE), CINAHL, EMBASE and Web of Science. In addition, reference lists of included studies were manually searched to find other potentially relevant citations, and forward reference searching was used to identify potential articles citing the included studies. Our search encompassed studies published in English up to 31 December 2018. No lower limit was used to restrict publication date.

The search strategy contained synonyms and sub-topics—in the form of database-specific subject headings as well as free terms—of the following concepts: *post-mortem; health-related and genetic data; research ethics; stakeholders; preferences*. These synonyms were discussed among the research team beforehand in order to facilitate finding all relevant papers, such as those discussing different types of stakeholders (e.g., research participants, relatives and the general public). The full search strategy is available in Supplementary Table S1 online.

### Eligibility criteria

The list of retrieved studies was first screened on the basis of title and abstract, and subsequently by reviewing the full text of each of the articles selected during the previous round. Screening was done independently by two researchers (HA and MARB) who discussed the results until an agreement was reached. Studies were considered to be eligible for inclusion if they: (a) discussed the sharing or (re-)use, after the research participant’s death, of genetic and health-related data obtained while the participant was still alive; (b) explored the perspectives or experiences of stakeholders regarding this process; (c) were empirical in nature. We chose to include both quantitative and qualitative studies, in order to enumerate acceptability levels while also enabling understanding of underlying reasons.

Articles were excluded if they were: (a) a systematic review; (b) an editorial, letter, case description or other type of descriptive article with no empirical data; (c) a discussion of attitudes towards data being used for clinical or forensic purposes instead of research; (e) a description of perspectives on the use of data obtained *after the death* of the research subject; (d) not written in English. No exclusions were made based on age, sex, ethnicity or any other characteristic of participants.

### Data extraction and quality appraisal

Data extraction was done independently by two researchers (HA and MARB) who both used the same standardised form that was developed for this review. Only findings related to use of data after death were extracted, which were only a small part of the results in some studies. The resulting summary tables were used as a basis for Table 1 of this article. Findings were reported according to the Preferred Reporting Items for Systematic Reviews and Meta-Analysis (PRISMA) statement [31]. We did not perform a meta-analysis given the variety of research questions and study designs.

To assess the risk of bias and overall validity of the articles, we used the Joanna Briggs Institute tools for qualitative research and for analytical cross-sectional studies, the latter when appraising survey studies since no specific tool existed for survey methods [32]. These tools consist of 8 to 10 questions, where a higher score indicates a lower chance of bias. Quality appraisal was done by one researcher (HA) and discussed with a second researcher (MARB) for validation of the assessment. Insight into study quality was important in interpreting results, but we decided that no articles would be excluded based on quality, given the expected scarcity of literature.

## Results

### Included studies

**Fig. 1:**
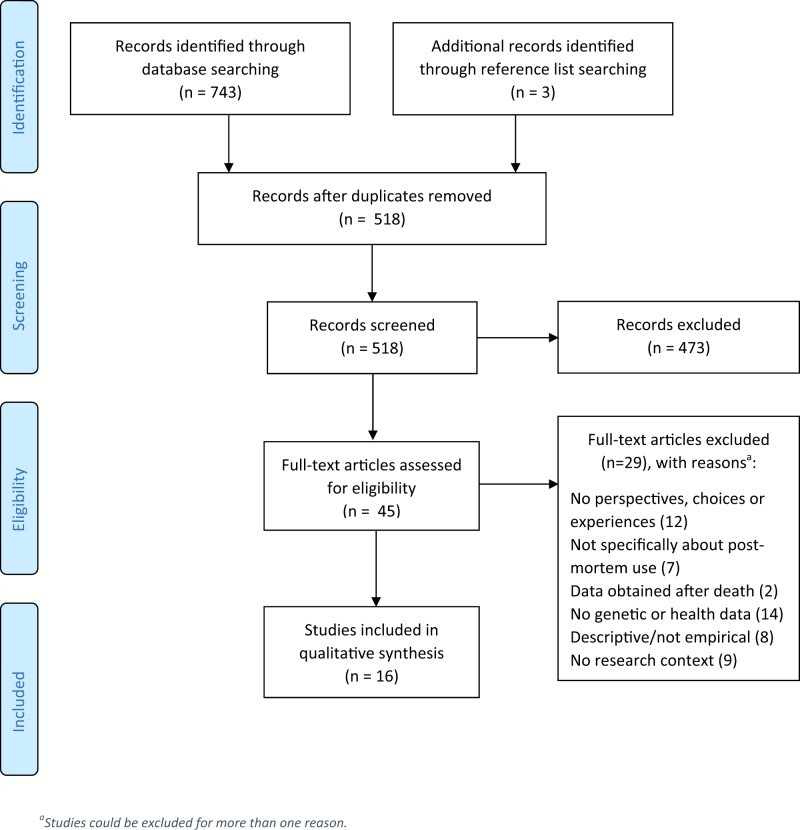
Flow diagram depicting the search and screening procedure

As shown in the PRISMA flow diagram, through our systematic search we identified 518 studies of which 16 were included in this review (Fig. 1). Included studies were published between 2007 and 2018 and were from North America (*n* = 11), the United Kingdom (*n* = 2), Poland (*n* = 1) and Australia (*n* = 2). Data collection dates were not reported in several studies. Stakeholders included living research participants (patients as well as healthy controls), relatives of deceased participants, researchers, IRB chairs and vice chairs. Studies included a total of ~5400 individuals who were mostly white and older than 40. Across the ten studies that reported gender, a slim majority of respondents (57%) was female. Perspectives, experiences or choices regarding the use of post-mortem data were explored using cross-sectional surveys, semi-structured interviews, and secondary analyses of consent documentation. The focus of all retrieved studies was on genetic data rather than (solely) on health-related data such as patient records. A number of included studies (*n* = 4) focused specifically on the use of data from paediatric patients. All articles scored relatively high on quality appraisal criteria, although it should be noted that in some studies, preferences regarding post-mortem use comprised only a subsection of the results. Characteristics of the included studies and study findings are shown in Table 1. In the following sections, results are presented according to the two main themes found in the reviewed studies: (1) perspectives on the post-mortem use of genetic and health-related research data, and (2) views regarding the return of individual genetic research results to relatives after the participant’s death.

**Table 1:** Characteristics and results of the included studies

Study ID	Country	Study design	Participant characteristics	Sample size (*n*)	Relevant outcomes measure(s)	Key findings	Quality appraisal
[33]	Australia	Secondary analysis of consent forms for use of samples.	Parents of deceased paediatric cancer patients whose tissue and data was available.	12	• Willingness to consent to research using their child’s tissue and data after death. • Desire to receive results.	• All parents (100%) consented to both current and future research projects. • 92% of parents consented to be notified if a genetic risk factor were to be found.	6/8
[34]	Canada	Self-administered paper or online survey.	Parents of children with cancer or orphan diseases who participate in research. Parents were 86% white and 87% was 35 years or older.	362	• Perspectives on the types of research purposes that previously collected tissue from children who died from cancer should be used for. • Perspectives regarding the sharing of research results after a child’s death.	• 21% felt tissue should be used only for original purposes, while 46.5% stated data should be used for whatever research necessary and 49% said parents should be contacted about their wishes. Only 3.5% thought that tissue should not be used for new research at all. • 76.5% thought it most appropriate to share data with parents or next of kin. According to 8%, results should be solely published in medical literature.	7/8
[35]	Poland	Self-administered paper survey.	Biobank managers. No characteristics provided.	24	• Whether biological samples of biobank participants can be used after their death.	• 83% of biobank managers supported the use of deceased participants’ samples if they had not given any written objection to this whilst alive.	5/8
[36]	Australia	Semi-structured interviews.	Parents of children whose samples were taken for diagnosis of mitochondrial disorder and later used for research. Parents were aged between 28 and 58 years.	9	• Opinions about being re-contacted with new genetic research results years after the death of their child. • Reactions to these new research results and attitudes towards its usefulness.	• All were pleased research was ongoing. None had objected to being re-contacted, some had actively contacted the researchers. Two families found the hospital visit emotionally difficult. • Whether they found results important, depended on previous beliefs about the disorder. Difficult knowing how and when to inform other relatives. Most parents told surviving children, some told extended family, if seen as relevant.	8/10
[38]	USA	Self-administered electronic or paper survey.	OurGenes Biobank participants who were 34% male, 87.4% white, mean age 56 years.	555	• Opinions on whether post-mortem genetic research results should be shared. • Attitudes regarding with whom it should be shared.	• 9% would not want to share their genetic results with anyone at all. • 52% would want data to be shared with blood relatives and 30% with someone not related by blood.	7/8
[39]	USA	Secondary analysis of preferences recorded during trial consent procedure.	Patients evaluated for hereditary colorectal cancer and/or polyps, who were 58% male, 80% white, mean age 52 years.	78	• Choices regarding whether to disclose research results to relatives after death. • Choices regarding the person selected to whom the results will be disclosed.	• 92% of participants agreed to having their data shared with family members. • Reasons for declining: no (relationship with) relatives; uncomfortable sharing unknown data; privacy concerns; not wanting to burden someone with the information. • Designated receivers were spouses (65%) or blood relatives (35%) who were mostly female. Participants with young children mostly chose their spouse. Participants considered whether the designated person would likely share results with others at risk.	7/10
[40]	USA	Self-administered paper survey, using the same hypothetical situation as [45].	Patients in a pancreatic cancer biobank, their spouses and blood relatives, and healthy individuals as control group. Participants were 43% male, 98% white, mean age 66 years.	3630	• Preferences regarding whether results are to remain private after death. • Attitudes on whether it is permissible to share Pat’s data with a spouse or blood relatives after death (in three gene variant scenarios). • Attitudes regarding who should decide after death of the patient.	• 4.4% of patients and relatives and 6.5% of controls would not want genetic research data to be disclosed after their death. • A majority agreed that results should be offered to Pat’s spouse (around 90%) or adult children (around 70–75%). If Pat had not wanted to share results with relatives, one-third said this wish should be honoured. • 39% said spouses should make the decisions, and 36% chose a blood relative. Researchers and primary care providers were, respectively, chosen by 7% and 8% of respondents.	6/8
[41]	USA	Semi-structured interviews (supplemented with survey data reported in [45].	Pancreatic cancer biobank participants and family members. Participants were 98% white, 47% male and median age 55–68.	51	• Acceptability of the return of research results to relatives after death. • Attitudes on who should control access to deceased participants’ results.	• 98% of interviewees agreed that genetic results obtained posthumously should be offered to relatives. Respectively 34% and 32% found sharing acceptable regardless of the deceased’s wishes or when these wishes were unknown. 22% thought permission of the deceased person or their representative was needed. The only factor that influenced preferences was the uncertainty of results, not the preventability and treatability of a condition. • Most interviewees thought participants should be asked about post-mortem return of results when entering a biobank or registry, but some said participants choices should not be decisive, and felt there is a moral responsibility to share with other relatives, and that genetic data belongs to all family members.	9/10
[42]	USA	Secondary analysis of survey data from [40].	Population from [40], without controls. 37% male, 98% white, mean age 64 years.	1903	• Whether attitudes towards sharing of data after death were discordant between patients and family groups.	• There were very few attitudinal differences, with confidence intervals overlapping.	6/8
[37]	UK	Semi-structured interviews (either face to face or by telephone).	Relatives who had accepted an offer to receive results about now deceased participants in genetic research.	13	• Impact of being informed about genetic research results after the participant’s death.	• Reasons for accepting were interest and a sense of duty towards the deceased relative. Some were pleased to be contacted, for others it caused distress. Several participants did not seek genetic counselling after finding out the results. When asked, participants would not prefer their relative’s data to have been anonymised.	8/10
[43]	Canada	Self-administered paper survey.	Genomic researchers, of whom 18% was older than 55 years and 54% between the ages of 41 and 55.	74	• Attitudes on whether genetic results of children, who died before results were available, should be offered to relatives.	• 81% stated that research results should be returned to parents or next of kin, without specific instructions prior to death.	8/8
[44]	Canada	Self-administered electronic or postal survey, companion study to [45].	Research Ethics Board members who were 32% male, most over 50 years of age and white.	22	• Opinions on whether a deceased (hypothetical) cancer patient’s family should be offered genetic results. • Opinions on whether deceased participants’ preferences should be followed. • Opinions on whether relatives should be offered results in a non-disease-specific context.	• 27% said results should probably be returned to family. When the consent form had been proactive in contemplate sharing of findings with relatives, 36% would *not* disclose to family. • If the participant had been asked about disclosure to family and had said “no”, 77% stated relatives should not receive results. • In a general context, 45% said there are circumstances in which genetic results are offered. Important considerations were consent statements, clinical validity, clinical utility and seriousness of the disease.	6/8
[45]	USA	Self-administered electronic survey, focused around the hypothetical scenario of cancer patient “Pat”.	Institutional Review Board members who were 55% male, 88% white, 75% older than 50.	65	• Opinions on whether deceased (hypothetical) patient Pat’s family should be offered genetic results. • Opinions on whether participants should be asked to make choices about family receiving results after death. • Opinions on whether relatives should be offered results in a non-disease-specific context (i.e., not Pat).	• When the consent form stated data could be returned when relevant: 25% said results should be returned to Pat’s family, 58% said they should not, 15% was unsure. If the consent was silent on this topic, 51% said this would have no effect on their decision. When the consent form states that data is returned to the participant, 55% would not disclose to family. • 62% said that participants should be asked to indicate choices regarding disclosure. If Pat had been asked and had said “no”, 88% stated relatives should not receive results. • When no specific disease was named, 51% said relatives can be offered results. Consent statements, clinical validity and seriousness of disease were deemed important considerations.	6/8
[46]	USA	Self-administered electronic survey and semi-structured telephone interviews.	Genomic researchers: 64% male, 73% white, mean age 43 years. Research subjects with congenital disorders: 15% male, 85% white, mean age 44 years.	Survey: 241 researchers. Interviews: 28 researchers, 20 patients.	• Opinions on whether participants should be informed during the consent process about how individual findings will be handled after their death.	• 64% of researchers surveyed and 90% of participants interviewed, indicated the subject should be informed at the time of initial consent. One researcher feared this could result in the patient not partaking in the research.	7/10
[47]	USA	Secondary analysis of question asked in trial consent procedure, same as [39].	Patients evaluated for hereditary colorectal cancer and/or polyps, who were 54% male, 85% white, 64% older than 50.	61	• Choices regarding the person to be the estate executor and the person authorised to receive genetic results.	• 69% had an estate plan, of whom 33% chose a different designee for genetic research results than their estate executor.	7/8
[48]	UK	Separate analysis of one subtheme that arose from the interviews in [37].	Same population as [37].	13	• The process of dissemination of a deceased relative’s result within families. • The experience of being informed.	• One person initially acted as informer. Older family members were told, who then passed on the information to children. In the wider family, people were unsure who had been told. • Difficult family relations inhibited sharing. Results offered through relatives were not always clear, which could be distressing. Several participants preferred to be contacted directly.	8/10

### Perspectives on post-mortem use of data for research

#### Should researchers be able to use data from deceased participants?

Four studies were identified that reported attitudes (only among family members and researchers) towards research being conducted after the death of the research subject [33–36]. Overall, acceptability of post-mortem use of previously collected data for research was high. In an Australian study, all parents (100%) of deceased paediatric cancer patients were willing to consent to current and future research projects with data of their child [33]. In another study of parental attitudes, conducted in Canada, 96.5% of respondents found it acceptable that their child’s previously collected tissue was used for genetic research posthumously, with views ranging from only allowing research use for original purposes (21%) to whatever research necessary (46.5%) [34]. A small survey study among Polish biobank managers found that 83% supported the use of genetic data after death if no written objection had been given whilst alive [35]. A fourth study used interviews to explore factors influencing acceptability, as discussed hereafter [36].

#### Factors underlying perspectives toward post-mortem research

In a qualitative study assessing experiences of Australian parents whose departed child’s genetic data was used for secondary research, the news about ongoing research was favourably received [36]. Parents wanted to spare others the pain and grief they suffered, as one participant described when asked whether she wanted to be contacted about genetic research results:

*“Absolutely. Look, to me, she died, she was beautiful, there was nothing anyone could do, there was nothing I could do, and if having her tissue, or whatever they had of hers was going to help one child be diagnosed quicker than what she was… You know, to save the parents that terrible anguish of not knowing, or to, you know, to find out more about it, I was happy for that to happen.” (p. 265)* [36]

The desire to help future research and patients was named as a major factor that shaped their positive attitudes towards the use of data for research purposes [36]. In addition to this sense of altruism, parents were pleased to find out about implications for their surviving family members.

#### Who should decide about usage of data for research after death?

One Canadian study investigated whether relatives of deceased participants (in this case: parents) should be contacted about their wishes regarding post-mortem research use of data, and found that approximately half (49%) thought they should indeed be asked for consent regarding new research uses [37]. Most included studies, however, only reflected on consent for post-mortem use of research data that personally affect *living persons* as well, i.e., in the context of disclosure of potentially actionable genetic findings to relatives.

### Views on post-mortem return of individual genetic research findings to relatives

#### Should individual genetic findings be shared with relatives of deceased participants?

Most studies focused on the familial implications associated with post-mortem genetic research data—rather than on the use as such. The return of genetic findings with potential clinical relevance for relatives was discussed in 15 out of 16 studies [33, 34, 36–48]. The overall majority (ranging between 76.5 and 98%) of research participants and family members believed that individual genetic findings should be shared with relatives after the participant’s death [33, 34, 39–41]. Attitudes did not differ significantly between participants and relatives, as seen from analysis of US survey results [42].

Fernandez et al. [43] surveyed Canadian genomics researchers most of whom (81%) believed that individual research results should be offered to relatives, if there were no specific instructions prior to death. In contrast, only about half (45% in Canada and 51% in the United States) of IRB chairs and vice chairs said that under certain circumstances relatives can be offered results of a deceased research participant, that is, when no specific disease was mentioned [44, 45].

#### To share or not to share: relevant factors in deciding to return post-mortem results

When IRB members were asked instead about the hypothetical scenario of Pat—a deceased pancreatic cancer patient whose DNA researchers discovered had a variant of unknown clinical significance—only a minority (27% in Canada and 25% in the United States) believed results should be returned to Pat’s family [44, 45]. In addition to clinical utility, which is lacking in the case of Pat, other relevant factors according to IRB members were the seriousness of the disease, clinical validity of the results and the type of statements made in consent forms [44, 45]. Contrary to this, biobank participants and relatives in a study from the United States did not consider preventability or treatability as factors in deciding on the return of results, but only regarded the (un)certainty of results as important [41].

Analysis of consent preferences in a US study found that research participants’ reasons to object to the sharing of findings in the event of their death included: having no relatives or not a good relationship with them, privacy concerns, and being uncomfortable burdening family members with potentially distressing information [25]. Relatives’ reasons for preferring to receive genetic findings were found to be a sense of duty towards their deceased family member as well as their own interest in genetic knowledge in order to plan for the future [37, 41].

#### Experiences with receiving post-mortem individual genetic research findings

Despite wanting to know the results, some parents experienced emotional distress from visiting the hospital (to discuss genetic findings) for the first time since their child’s death as well as from the potential impact on their other children:

*“…what I thought had been laid to rest, at least in that department, has been reopened, by further knowledge. […] Yeah, which is important to have, but difficult to deal with.” (p. 267)* [36]

Relatives experienced distress especially when information relayed through family members was unclear. Moreover, when only one or a small number of relatives was informed, downstream communication of the results could be inhibited, e.g., by problematic family dynamics [36, 48]. In one interview study from the United Kingdom, participants indicated that they would have preferred being contacted by the research team directly instead of through family [48].

#### Who should decide about the post-mortem return of individual genetic research findings?

Three US-based studies explored who stakeholders thought the designated person to receive individual results should be, and found that research participants would either choose biological relatives (35–52%) or someone not related by blood such as a partner (30–65%) as the designated person to be offered results [38–40]. When given the option, only a minority of participants selected researchers (7%) or primary care providers (8%) as the appropriate person to make decisions about the return of results [40]. Notably, in reality about a third (33%) of participants in another US study designated a different person for receiving genetic results than the person who would normally receive the results according to state law [47]. Had this choice not been provided, the person deemed most appropriate to decide about further disclosure would not be the one actually receiving them.

A majority of stakeholders (62% of IRB members, 64% of researchers and 90% of research participants, all from the US) indicated that research subjects themselves should be informed or asked about post-mortem disclosure of findings to relatives *during the initial consent talk* [45, 46]. Nonetheless, one researcher noted this might be a delicate issue and could prevent people from taking part in research:

*“I think the issue of what to do if someone dies is an important but difficult question. Since I work with cancer patients, often at the time of diagnosis they are not ready to talk about what happens if they die, and I could see having this discussion could easily cause them to become angry and not enter the study.” (p. 370)* [46]

#### Can the deceased’s preferences be overridden?

An important question is whether a person’s preferences can be overridden after death. This was discussed in three studies originating from the United States [40, 41, 46]. A large majority (88%) of IRB members said relatives should not receive individual research results if the research participant had indicated not wanting these to be shared after death [46]. Between one-third and two-thirds of research participants and their relatives thought this wish to keep findings private should be respected [40, 41]. A number of biobank participants and relatives believed confidentiality should not be promised during the consent process:

*“I know there are people who don’t want their families to know, and so then that is a tough question. First of all, if you discussed that with a patient who had the cancer, but then if they said ‘I don’t want anybody else to know’, then I probably think that you should tell them, ‘Well, we feel it is necessary [to let your family know]’.” (p. 14)* [41]

However, interviewed research participants and family members were divided on this issue, with some stating that the data *“should be private unless that person, while they are alive, grants permission”* and others noting that *“a dead person is no longer a person and therefore has no rights”* [41].

## Discussion

This literature review is the first to systematically synthesise the empirical literature on the ethical questions around post-mortem use of data for medical research. All studies were published in the last decade, signifying increased awareness of the topic likely correlated with the advancements in DNA sequencing. Both quantitative and qualitative methods were found to be used in ascertaining the views and experiences of patients, relatives, healthy controls, researchers and IRB members about (1) the use of research data after death, and (2) the post-mortem return of individual genetic research results to relatives. We discuss these themes in turn and conclude with some reflections on consent and the desired extent of control regarding post-mortem use of data for medical research.

### Post-mortem use of data for research: an underexplored area

Regarding the question of acceptability of post-mortem use of previously collected data for research, we found limited evidence. The four identified studies showed high levels of support among relatives and researchers (no studies were found eliciting views from participants themselves) for using data from the deceased for research purposes. This is in line with the views of living research participants who generally support research using clinical data or samples, and believe the contribution to research outweighs the risks of re-identification or unwanted uses [49].

Compared with living participants, for the deceased this balance between benefits and risks may be further skewed towards the benefits, given the diminished possibility of harm from data breaches for deceased participants. On the other hand, there seem to be risks for deceased subjects too, given the potential harm to their ante-mortem integrity or changes to their legacy which they cannot correct [50]. While the philosophical debate [51] on whether dead people can be harmed deserves further attention, a full discussion is outside the scope of this article. Of note is that *genetic* data brings about additional risks in terms of the potential implications for blood relatives sharing genetic traits with the deceased, namely potential practical concerns (e.g., insurance and employment) and psychological harm associated with clinically actionable findings.

In our analysis of empirical studies we found a complete lack of research regarding health-related data outside of genetics, probably because these implications of post-mortem use are most obvious in relation to genetic relatives. However, the focus on genetic data obscures the question whether research participants should have post-mortem informational self-determination as such, independent of whether their data have health implications for their relatives [52]. This question warrants further attention and studies are needed that focus on other types of health data.

### Return of individual research findings to relatives after death

The majority of studies included in this review focused on the post-mortem sharing of genetic findings of potential clinical relevance with relatives. In considering whether to return these findings, rights of the deceased ought to be weighed against those of living relatives interested in receiving such findings (in particular, the right to know; which itself may conflict with interests of other relatives who do *not* want to know). Boers et al. [53] have provided an overview of arguments in favour of and against post-mortem disclosure of genetic information to relatives. Our results suggest that of those arguments, reasons of participants and family members for not supporting post-mortem disclosure are mostly related to the principles of respect for privacy of the deceased and of non-maleficence, i.e., the wish to avoid the harm of emotionally burdening relatives with these individual research findings. Professionals (researchers and IRB members) were less supportive of post-mortem disclosure of genetic findings than lay persons (participants and their relatives). No qualitative explanation was found in the empirical literature, but possible reasons could be professionals’ greater experience with the ethical difficulties related to genetic findings and a recognition of relatives’ right not to know, as well as insight into the costs and time involved in offering results to family.

Questions regarding disclosure of individual genetic research findings to relatives are not specific for the situation after death and, similar to the context in which the participant is alive, consensus in the literature seems to be that findings can be offered if they are analytically valid, likely to be of substantial clinical significance for relatives, and actionable [28, 54]. Knoppers et al. have concluded that there exists an ethical duty for researchers to return such results (“duty to warn”) to participants, that is, while leaving room for the right not to know [55].

In the setting where the participant has died, this duty seems less obvious given the lack of relationship between researchers and participants’ relatives [28]. Therefore, in the context of post-mortem disclosure of genetic information to relatives, several authors have advocated a less proactive (“passive”) disclosure policy meaning that findings are returned only upon the request of family [28, 53, 54]. Only in very exceptional cases of high pathogenicity would the researchers initiate return (i.e., active disclosure). Such a policy would preserve the relatives’ interest in not knowing, and would minimise the burden on the research enterprise thereby leaving more resources to be used for studies that benefit future patients. Yet other authors have stated this may not be sufficient and that active disclosure policies should be explored, given the moral requirement of beneficence and the known barriers to downstream communication of post-mortem genetic findings among surviving relatives [56].

While debatable, it has been suggested that researchers could help manage these barriers through offering of ancillary care such as supporting downstream communication of results, e.g., by supplementing family mediated disclosure with genetic counselling, while taking account of the effect on familial relationships [48, 54, 57]. Concrete tools and context-sensitive disclosure guidance are needed to aid researchers in the disclosure process and provide suggestions on issues like which relative(s) to share results with and how to handle family conflict. One example of a pragmatic tool for researchers, developed in the United States by Wolf et al. [58], is based on the situation where a personal representative decides on the sharing of results with surviving relatives, which, however, may not be regarded as an appropriate pathway and level of control in every context [59].

### Informing participants about post-mortem uses of research data

Potentially, when asked, participants would consent to continued research use of their data after death, and some authors have stated that it would be reasonable to presume an altruistic intent [60]. However, in many studies this continued post-mortem use of data is currently *not made explicit* during consent procedures [4–7], although there are exceptions [61], and it has been recommended in rare disease research that the destination of and access to post-mortem data should be included in informed consent documents [62]. The Organisation for Economic Cooperation and Development, in their Guidelines on Human Biobanks and Genetic Research Databases, also recommends biobanks to include policy provisions on the use of data after death, which should be made explicit to participants during the informed consent process [63]. Echoing these recommendations and stakeholders’ perspectives as included in our review, we argue that researchers should have policies in place around post-mortem use of data and discuss these with potential participants during initial consent.

The ethical rationale for informing people about the future uses of their data, even after death, is twofold. Firstly, this is important from a *deontological* (i.e., duty-based) perspective, which recognises data privacy as valuable in itself, and information provision about future research uses (including after death) as a way to respect participants’ autonomy. Secondly, the *utilitarian* (i.e., consequence-based) argument is that transparency is needed for maintaining public trust in science, as the success of biobanks and registries depends on people’s willingness to participate in research, which in turn is important to promote the public’s health.

As Jessica Berg has noted, these consequences of not informing participants about post-mortem use are difficult to ascertain. On the one hand, not discussing post-mortem use of data during consent talks is current practice in most studies, and this lack of transparency currently does not seem to compromise research participation [64]. However, the increased interest in privacy following the introduction of the GDPR in Europe might lead people to start questioning their own assumptions and the regulations regarding post-mortem privacy. For instance, the public may be unaware that in many jurisdictions, it is not the relatives who decide whether data of the deceased may be used post-mortem for research. Moreover, sharing of genetic findings with relatives is generally seen as a benefit of research participation by both living participants [54] and parents of deceased participants [36], and participants may expect that clinically actionable results are shared with relatives after their death, which is not necessarily the case. Informing prospective participants about both post-mortem research uses of data and the disclosure of individual findings, if possible, would demonstrate that researchers take their responsibilities seriously, and knowing that participants have considered this issue whilst alive would remove some uncertainty when balancing interests after their death [30].

Whether this obligation to inform also holds with regard to the deceased’s relatives, requires further research. When blood relatives were previously unaware that the deceased person’s data is posthumously used without consent, hearing about this can cause distress [37] and, it has been suggested, potentially anger or mistrust towards research in general [65]. Yet, contact with relatives is often logistically problematic, and reaching out to grieving people requires a sensitive approach. Thus, potential negative consequences of informing participants or relatives about the use of research data after death should be considered too, not only because death may be a distressing topic, but also because it is currently unclear whether such information would deter potential participants.

### Different views regarding control over post-mortem research data

With regard to the question whether participants or relatives should not only be *informed* but also have the authority to *control* and make decisions about use and sharing of post-mortem data specifically (either regarding research uses or return of individual genetic findings to relatives), our findings do not provide a straightforward answer. The European Society of Human Genetics has stated that restrictions placed by living research participants on the research use of genetic samples should continue to apply after death [66], although elsewhere they recommend that the use of *anonymised* genetic samples and data from the deceased should be allowed for research purposes, and that access to the deceased’s genetic data could be legitimate in case of overriding interests of blood relatives [67]. Likewise, most legal systems internationally have provisions to break confidentiality when it is necessary to serve important (“legitimate”) health interests of relatives, e.g., to warn them in case of risks from genetic diseases [68]. However, disclosure policies have also been proposed, in which participants can choose not to release results to family members when they die [69].

Further research is needed into the desirability of allowing control by participants or (specific) relatives, and on the question whether the deceased’s wishes can be overridden, while taking into account what is possible in view of existing national (case) law. Some authors have stated that, when no decision has been made by the participant, the responsibility for these assessments should lie with relevant authorities such as IRBs [60, 65]. Yet, the reviewed empirical literature suggests that this may not be desirable given the aforementioned discrepancy in views between participants and IRB members, which calls for further exploration.

It is worth mentioning an alternative to post-mortem research without specific consent that has been gaining traction in recent years, namely, one in which the general public is explicitly asked to think about post-mortem data use. Several authors have advocated the establishment of so-called posthumous medical data donation registries, where people indicate their preferences in a manner analogous to organ donation schemes [70, 71]. This approach would be autonomy-enhancing for participants, eliminate the need for others to make these decisions, and would be useful for studies looking to use data and samples from incapacitated patients who cannot be asked to consent. However, as studies have shown that diseased persons are more likely to accept use of data for research compared to healthy individuals [49], we hypothesise that asking people to register their preferences outside of a medical study context may lead to refusals and in turn, lack of generalizability of the acquired data. In addition, questions would remain around responsibilities of returning findings to genetic relatives.

### Limitations

Our study is not without limitations. To start, the number of articles that met inclusion criteria was relatively small, and several included studies only minimally considered the situation after death. Studies were not only few, but also limited to the field of genetics, as discussed above. While our search strategy encompassed all types of health-related data, retrieved studies only considered genetic data and mostly in the context of returning actionable findings. Second, comparability of included studies may be limited. This is due to questions being differentially worded, which slightly changes their meaning (e.g., “should vs. may” results be “disclosed vs. offered vs. returned” to “relatives vs. next-of-kin”?). Comparison between studies was also potentially limited because while some authors investigated actual choices made, others used hypothetical scenarios to gauge people’s opinions. Third, our findings may lack generalizability both across and within studies. Limiting the search to articles published in English has produced mostly studies from North America, some parts of Europe and Australia, while cultural attitudes towards death and the use of data or material, as well as already existing legal and ethical frameworks may differ widely internationally. Within included studies, bias may have been introduced by the substantial overrepresentation of white people over 40 years old, the small sample sizes, specific settings (i.e., mostly cancer research), and the fact that individuals choosing to participate in these studies may already have positive attitudes towards research. Future studies should involve larger and more diverse groups of participants in terms of age, ethnicity, nationality and setting.

## Conclusion

This literature review provides evidence of high acceptability of post-mortem use of data for medical research, while highlighting knowledge gaps and targets for policy improvement. In order to prevent a move in one of two opposite and equally autonomy-limiting directions, namely either paternalistic overprotection or blanket presumed consent for post-mortem use of data, there is a strong need for further legal and ethical guidance on this topic. Such guidance should also address the topic of returning individual genetic research findings to relatives of the deceased.

## Supplementary information

Table S1 - Search strategy
