# Supplementary material for: Stakeholders’ perspectives on the post-mortem use of genetic and health-related data for research: a systematic review
Source: Eur J Hum Genet. 2019 Sep 16;28(4):403–16. doi: 10.1038/s41431-019-0503-5 (PMC7080773; doi:10.1038/s41431-019-0503-5)
Supplement: Supplementary file 1 — Table S1 - Search strategy [file 41431_2019_503_MOESM1_ESM.docx]

**Supplementary material**

Table S1. Search strategy (shown here is the PubMed version as example).

| **Set#** | **Search string** | **Results** |
| --- | --- | --- |
| 1 | "Registries"[MeSH] OR "health data" OR "health information" OR "Electronic Health Records"[MeSH] OR biobank*[tiab] OR bio-bank*[tiab] OR biorepository*[tiab] OR "Databases, Factual"[MeSH] OR registry[tiab] OR registries[tiab] OR databank*[tiab] OR genomic*[tiab] OR "genetic research"[tiab] OR genetic*[tiab] OR "individual finding*"[tiab] OR "genetic finding*"[tiab] OR "Genetics"[MeSH] | 1 530 689 |
| 2 | "Privacy"[MeSH] OR "Confidentiality"[MeSH] OR "Personally Identifiable Information"[MeSH] OR privacy*[tiab] OR confidential*[tiab] OR "Informed Consent"[MeSH] OR “informed consent”[tiab] OR "Ethics, Research"[MeSH] OR "Patient Rights"[MeSH] OR disclos*[tiab] OR "Information Dissemination"[MeSH] OR "Communication"[MeSH] OR communicat*[tiab] OR "Duty to Recontact"[MeSH] | 668 824 |
| 3 | Patients"[MeSH] OR "Stakeholder Participation"[MeSH] OR famil*[tiab] OR relative*[tiab] OR participant*[tiab] OR population[tiab] OR public[tiab] OR community[tiab] OR societ*[tiab] OR "Research Subjects"[MeSH] OR researchers[tiab] OR institutions[tiab] | 4 590 151 |
| 4 | "Death"[MeSH] OR deceased[tiab] OR death*[tiab] OR departed[tiab] OR died[tiab] OR dead[tiab] OR post-mortem[tiab] OR postmortem[tiab] OR posthumous[tiab] | 1 079 738 |
| 5 | opinions[tiab] OR perspectives[tiab] OR views[tiab] OR experiences[tiab] OR viewpoint*[tiab] OR willingness[tiab] OR preference*[tiab] OR attitude*[tiab] OR impact[tiab] OR choice*[tiab] OR support[tiab] | 2 423 868 |
| 6 | (#2 AND #3 AND #4 AND #5 AND #6 AND English[Language]) | 281 |
